# P-6. Effect of a school-based tutorial about the HPV vaccine for female middle school students on the HPV vaccination rate in Japan

**DOI:** 10.1093/ofid/ofae631.217

**Published:** 2025-01-29

**Authors:** Yo Murata, Yuya Saito, Toshimasa Obonai

**Affiliations:** Tokyo Metropolitan Children's Medical Center, Fuchu, Tokyo, Japan; Tokyo Metropolitan Tama-hokubu Medical Center, Higashimurayama, Tokyo, Japan; Tokyo Metropolitan Tama-hokubu Medical Center, Higashimurayama, Tokyo, Japan

## Abstract

**Background:**

Vaccine hesitancy related to the HPV vaccine poses a global challenge. In Japan, the Ministry of Health, Labour and Welfare (MHLW) rescinded recommendations for the inclusion of the HPV vaccine in the national immunization program following reports of adverse events in 2013, which led to a drop in the vaccination rate to less than 1%. Despite the MHLW's decision to reinstate the recommendations in 2022, the vaccination rate has remained low partly because the information targeting children and their guardians is insufficient. This study aimed to assess the effect of a school-based vaccine tutorial on the HPV vaccination rate in female middle school students in Japan.

**Figure 1:**
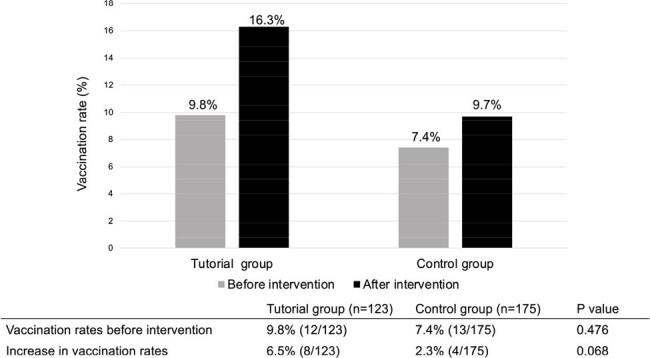
Difference in the pre- and the post-intervention vaccination rate.

**Methods:**

This prospective study, conducted in Higashimurayama, Tokyo from September 2023 to March 2024, enrolled female students aged 13 to 14 years in seven middle schools. Four and three schools formed the tutorial group and control group, respectively and information about HPV vaccination was provided to the tutorial group at school. The difference in the pre-intervention vaccination rate (September 2023) and the post-intervention vaccination rate (March 2024) was assessed using a questionnaire. Additionally, HPV vaccine knowledge and the rate of students’ discussing the vaccine with their family were compared.

**Table 1:**
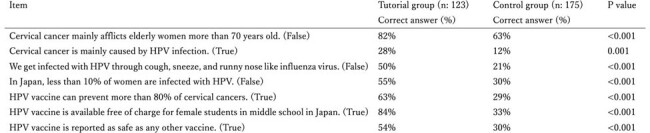
Knowledge about cervical cancer, HPV infection and HPV vaccination among tutorial group and control group.

**Results:**

In total 123 and 175 students from the tutorial and control group, respectively, were analyzed. The pre-intervention vaccination rate was 9.8% and 7.4% (p=0.476), and the post-intervention vaccination rate was 16.3% and 9.7% for the respective group, indicating an increase of 6.5% and 2.3%, respectively (p=0.068). (Figure 1) Knowledge about the HPV vaccine increased to a significantly greater degree in the tutorial group (Table 1), and the rate of students’ discussing the vaccine with their family was significantly higher in the tutorial group after the intervention (46% vs 28%; p=0.0018).

**Conclusion:**

Although the HPV vaccine tutorial did not cause a statistically significant increase in the vaccination rate, it demonstrated an upward trend in the tutorial group. Furthermore, the tutorial increased knowledge and interest in the vaccine among both the students and their family, suggesting that HPV vaccine tutorials can improve the HPV vaccination rate.

**Disclosures:**

**All Authors**: No reported disclosures

